# Frailty profile in Brazilian older adults: ELSI-Brazil

**DOI:** 10.11606/S1518-8787.2018052000616

**Published:** 2018-10-25

**Authors:** Juliana Mara Andrade, Yeda Aparecida de Oliveira Duarte, Luciana Correia Alves, Flávia Cristina Drumond Andrade, Paulo Roberto Borges de Souza, Maria Fernanda Lima-Costa, Fabíola Bof de Andrade

**Affiliations:** I Fundação Oswaldo Cruz. Instituto René Rachou. Programa de Pós-Graduação em Saúde Coletiva. Belo Horizonte, MG, Brasil; II Universidade de São Paulo. Escola de Enfermagem. São Paulo, SP, Brasil; III Universidade Estadual de Campinas. Departamento de Demografia do Instituto de Filosofia e Ciências Humanas. Campinas, SP, Brasil; IV University of Illinois at Urbana-Champaign. Kinesiology and Community Health. Champaign, IL, USA; V Fundação Oswaldo Cruz. Instituto de Comunicação e Informação Científica e Tecnologia em Saúde. Rio de Janeiro, RJ, Brasil; VI Fundação Oswaldo Cruz. Instituto René Rachou. Núcleo de Estudos em Saúde Pública e Envelhecimento. Belo Horizonte, MG, Brasil

**Keywords:** Aged, Aging, Health Status, Socioeconomic Factors, Health Surveys, Idoso, Envelhecimento, Nível de Saúde, Fatores Socioeconômicos, Inquéritos Epidemiológicos

## Abstract

**OBJECTIVE:**

To estimate the prevalence of frailty and to evaluate the associated factors in the non-institutionalized Brazilian population aged 50 years or older.

**METHODS:**

The analyses were conducted in 8,556 participants of the baseline survey of the Longitudinal Study of Health of the Brazilian Elderly (ELSI-Brazil) conducted in 2015 and 2016. Frailty was defined based on five characteristics: weight loss, weakness, slowness, exhaustion and low level of physical activity. Participants with three or more characteristics were classified as frail. A Poisson regression model was used to examine the association between frailty and sociodemographic and health factors.

**RESULTS:**

The prevalence of frailty was 9.0% (95%CI 8.0–10.1) among participants aged 50 years or over. Among the older adults aged 60 or over, the prevalence was 13.5% (95%CI 11.9–15.3) and 16.2% (95%CI 14.3–18.3) among those 65 aged years or over. Factors associated with higher prevalence of frailty were low schooling, residence without a partner, health conditions (poor self-rated health and two or more chronic diseases) and limitation to perform basic activities of daily living.

**CONCLUSIONS:**

The prevalence of frailty among Brazilians aged 65 years or older is similar to their European counterparts. Poor health conditions, functional limitation and low schooling emerge as the factors most strongly associated with the frailty in this population.

## RESUMO

**OBJETIVO:**

Estimar a prevalência de fragilidade e avaliar os fatores associados na população brasileira, não institucionalizada, com 50 anos ou mais.

**MÉTODOS:**

As análises foram conduzidas em 8.556 participantes da linha de base do Estudo Longitudinal da Saúde dos Idosos Brasileiros (ELSI-Brasil), entre 2015 e 2016. A fragilidade foi definida com base em cinco características: perda de peso, fraqueza, redução de velocidade de marcha, exaustão e baixo nível de atividade física. Os participantes com três ou mais características foram classificados como frágeis. As covariáveis incluíram características sociodemográficas e condições de saúde. As análises multivariadas foram realizadas por meio da regressão de Poisson.

**RESULTADOS:**

A prevalência de fragilidade foi de 9,0% (IC95% 8,0–10,1) na faixa etária de 50 anos ou mais. Na faixa etária de 60 anos ou mais, a prevalência aumentou para 13,5% (IC95% 11,9–15,3) e atingiu 16,2% (IC95% 14,3–18,3) entre aqueles com 65 anos ou mais. Os fatores associados à maior prevalência de fragilidade foram escolaridade mais baixa, residência sem um companheiro(a), condições de saúde (pior autoavaliação da saúde e duas ou mais doenças crônicas) e limitação para realizar atividades básicas da vida diária.

**CONCLUSÕES:**

A prevalência de fragilidade entre brasileiros com 65 anos ou mais é semelhante à observada, na faixa etária correspondente, em países europeus. As piores condições de saúde, a limitação funcional e a baixa escolaridade emergem como os fatores mais fortemente associados à fragilidade nessa população.

## INTRODUCTION

Frailty is characterized as an energy decline syndrome resulting from changes that occur due to aging. These changes predispose the elderly to a marked reduction in muscle mass and to a chronic inflammatory state, which, when associated with diseases, immobility or other extrinsic factors, results in a decrease in the energy reserve and an increase in physical vulnerability^1^. Different studies show that this syndrome is associated with advanced age^1^ ^-^ ^4^ and worse socioeconomic conditions, such as insufficient income and low educational levels^1^ ^,^ ^5^ ^,^ ^6^. Moreover, frailty is related to the presence of chronic diseases and disability, either by predisposing to them or resulting from them^1^.

In Brazil, the aging process occurs under unfavorable economic, social and health conditions^7^. This scenario creates conditions conducive to the development of serious health complications as people age^8^. In the last decade, due to the rapid aging of the population and the increase in the costs related to the health of the elderly, there has been an increase in scientific interest in the study of frailty^4^ ^,^ ^9^.

Population-based frailty studies in Brazil were conducted in cities previously selected as part of the Elderly Frailty (FIBRA) and the Health, Wellbeing and Aging (SABE)^4^ ^,^ ^9^ projects. Therefore, there are no studies to establish a national estimate of the prevalence of frailty and the factors associated with this condition. These factors deserve to be evaluated by virtue of the different conditions in which people grow old in the country. In clinical practice, evaluating and identifying the frailty syndrome in the elderly can help prevent the progression of this syndrome and minimize its adverse consequences^5^ ^,^ ^10^.

Knowledge about the epidemiological profile of frailty will allow future assessments of the impact of services and policies for the prevention and control of this syndrome. Thus, the objective of the present study was to estimate the prevalence of frailty and to evaluate the factors associated with this condition in a national representative sample of the population aged 50 years or older.

## METHODS

A cross-sectional study was conducted with data from the baseline of the Brazilian Longitudinal Study of Aging (ELSI-Brazil) conducted between 2015 and 2016. ELSI-Brazil is a prospective cohort study, conducted in a representative sample of the Brazilian population aged 50 years or older, living in 70 municipalities in the five major geographic regions of the country. This nationally representative sample used a multistate stratified cluster sampling design. All residents in the selected households, aged 50 years or older, were eligible for interviews, anthropometric evaluation, blood pressure measurements, and strength, balance and gait tests (n = 9,412). More details can be seen on the research website^a^ and previously published^11^ *.*

### Study Variables

#### Dependent variable

Frailty was defined as the presence of three or more of the following components: weight loss, weakness, low gait speed, exhaustion and low level of physical activity^1^. In the present study, each of these components was defined according to the criteria presented in a previous publication^12^.

Weight loss was assessed by self-report of weight loss in the last three months. Weakness was measured by the force of the hand grip using a manual dynamometer on the dominant upper limb. Each participant was asked to apply the highest possible force in three attempts, considering the best performance. Weakness was defined by the strength of the hand grip in the lower quintile [after adjusting for gender and body mass index (BMI) quartiles], as well as the condition of being bedridden and the inability to perform the test. The gait speed was measured by a timer to record the time spent (in seconds) to walk three meters, considering the smallest measure between two measurements. Low gait speed was defined by the highest quintile of time, stratified according to gender and height, as well as the inability to perform the test^13^. Exhaustion was defined by responses to the following questions from the Center for Epidemiological Studies’ (CES-D) depression questionnaire: “In the last week, how often did you feel that you could not carry things forward (started something but could not finish)?”; “In the last week, how often did your routine activities require a major effort to be completed?”. Exhaustion was attributed to those with frequencies greater than 3-4 days^14^. The physical activity score was calculated in metabolic equivalents per week and expressed in kilocalorie (kcal) based on the Short Form of the International Physical Activity Questionnaire (IPAQ)^15^. The IPAQ questions seek to evaluate the time (minutes and hours) and the intensity (mild, moderate and vigorous) of physical activity practices performed during the last week, based on work activities, to get from one place to another, in leisure, as a sport, as an exercise or as part of household chores^15^. Individuals in the lower quintile of expenditure in weekly kcal, stratified by gender, were considered to have a low level of physical activity. In the present study, the dependent variable was categorized as frail (three or more components) and not frail (two or fewer components). More details can be seen in a previous publication^12^.

#### Covariates

The covariates of this study included sociodemographic characteristics (age, sex, years of schooling, marital status, and perception of income sufficiency for household expenses), health behaviors and conditions [current tobacco consumption, self-rated health, multimorbidity, and ability to perform basic activities of daily living (BADL)]. Current smoker status was attributed to those who reported smoking daily. Self-rated health was assessed by the standard question ”How would you judge the state of your general health?”. The respondents used a five-point scale to answer, which were categorized into three groupsgood or very good, regular and poor or very poor. Multimorbidity was defined as the presence of two or more chronic diseases (*versus* one or none) as previously proposed^16^ ^,^ ^17^. The number of chronic diseases was defined by a history of medical diagnosis of the following diseases: hypertension, diabetes, heart disease, chronic lung disease, stroke, arthritis, asthma, cancer, and kidney disease. The variable functional disability was constructed by reporting any difficulty (unable, some or little) in performing one or more BADL, including bathing, dressing, feeding, using the toilet, getting out of bed, and crossing a room on the same floor^18^.

## Statistical Analyzes

The analyses of the associations between the independent variables and the outcome variable were based on prevalence ratios and 95% confidence intervals estimated using the univariate and multivariate Poisson regression. All the independent variables were included simultaneously in the final multivariate model. A Venn diagram was developed to describe the concomitance between frailty, BADL limitations, and multimorbidity (two or more chronic diseases). In addition, imputation analysis was performed using the MICE (multiple imputation using chained equations) procedure. A total of 10 imputed datasets were created to assess the influence of missing data on the estimates of the final frailty prevalence.For the imputation, the following variables were considered: age, gender, marital status and perception of income sufficiency. The analyses were performed using the Stata 14.0 program (Stata Corp., College Station, USA), using the svy command, which allows us to consider the complex structure of the sample, including the assignment of sample weights.

## Ethical Considerations

ELSI-Brazil was approved by the Research Ethics Committee of the Oswaldo Cruz Foundation, Minas Gerais (CAAE 34649814.3.0000.5091). All participants signed an informed consent form at the time of the interview.

## RESULTS

Among the 9,412 participants in the ELSI-Brazil baseline survey, 8,556 had complete information for all variables and were included in the present analysis. The average age of participants was 62.7 years (95%CI 61.9–63.5) and 53.4% were women. The prevalence of weight loss in the last three months, weakness, low gait speed, exhaustion and low level of physical activity were 7.4%, 22.6%, 20.5%, 28.6% and 19.8%, respectively. The overall prevalence of frailty was 9.0%. Other characteristics of study participants are shown in Table 1.

**Table 1:** Characteristics of study participants. The Brazilian Longitudinal Study of Aging (ELSI-Brazil), 2015-2016. (n = 8,556)

Variable	%	95%CI^a^
Age (years)		
50–59	48.7	44.6–52.8
60–69	29.8	27.9–31.7
70 or older	21.5	19.0–24.3
Female	53.4	50.4–56.4
Lives with a partner	64.8	61.8–67.6
Education (years of schooling)		
0–3	31.8	28.5–35.2
4–7	31.6	29.0–34.2
8–11	28.3	25.8–31.0
12 or more	8.3	7.2–9.6
Perceived income sufficiency		
Always enough	33.0	30.5–35.7
Sometimes it’s enough	26.1	24.4–27.8
Never enough	40.9	37.7–44.2
Current smoker	17.0	15.6–18.5
Two or more chronic diseases^b^	35.9	34.0–37.8
Difficulty performing BADL^c^		
No	85.5	84.1–86.7
Yes	14.5	13.3–15.9
Self-rated health		
Good	43.9	41.3–46.5
Regular	44.7	42.8–46.7
Poor	11.4	10.2–12.7
Frailty components		
Weight loss in the last 12 months	7.4	6.6–8.3
Weakness	22.6	20.7–24.5
Low gait speed	20.5	18.2–23.1
Exhaustion	28.6	26.5–30.8
Low physical activity	19.8	17.8–21.9
At least 3 of the above listed	9.0	8.0–10.1

^a^ Estimated 95% confidence intervals.

^b^ History of medical diagnosis of hypertension, diabetes, heart disease, chronic lung disease, stroke, arthritis, asthma, cancer, and kidney disease.

^c^ Difficulty in performing one of the following basic activities of daily living (BADL): bathing, dressing, eating, using the toilet, getting out of bed, crossing a room on the same floor.

**Figure 1:**
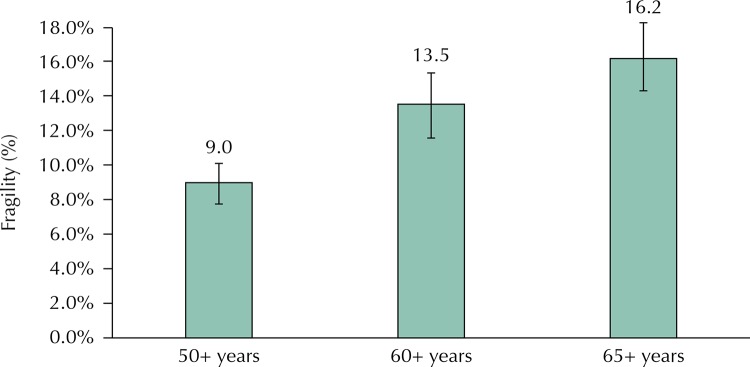
Prevalence of frailty according to age groups. The Brazilian Longitudinal Study of Aging (ELSI-Brazil), 2015–2016.

The prevalence of frailty gradually increased with age, from 9.0% (95%CI 8.0–10.1) in the age group of 50 years or older, to 13.5% (95%CI, 11.9–15, 3) in the age group of 60 years or older, and finally to 16.2% (95%CI 14.3–18.3) at age 65 or older (Figure 1).

The results of the unadjusted analysis (Table 2) show statistically significant associations between frailty and age, living with a partner, education, income sufficiency perception, self-rated health, multimorbidity, and difficulty to perform one or more BADL. Gender and smoking status were not statistically associated with frailty.

**Table 2:** Results of the unadjusted analysis of the association between frailty and sociodemographic characteristics, health behaviors and conditions. The Brazilian Longitudinal Study of Aging (ELSI-Brazil), 2015–2016.

Variable	Prevalence		Prevalence ratio	
	
	%	95%CI	PR^d^	95%CI^e^
Age (years)				
50–59	4.3	3.7–5.1	1	
60–69	8.1	6.9–9.5	1.87	1.49–2.34^b^
70 or older	20.9	18.1–24.0	4.82	3.99–5.83^b^
Gender				
Male	8.5	7.3–9.8	1	
Female	9.5	8.2–11.0	1.12	0.94–1.34
Lives with a partner				
No	11.6	10.2–13.0	1	
Yes	7.6	6.7–8.7	0.66	0.59–0.74^b^
Education (years of schooling)				
0–3	14.5	12.4–16.8	1	
4–7	8.4	7.3–9.7	0.58	0.47–0.71^b^
8–11	5.3	4.0–6.9	0.36	0.28–0.47^b^
12 or more	3.3	1.8–6.1	0.23	0.13–0.43^b^
Perceived income sufficiency				
Always enough	7.8	6.4–9.4	1	
Sometimes it’s enough	8.3	6.9–10.0	1.07	0.84–1.37
Never enough	10.5	9.2–11.9	1.35	1.14–1.61^c^
Current smoker				
No	9.0	7.9–10.2	1	
Yes	9.2	7.8–10.8	1.03	0.87–1.21
Self-rated health				
Good	4.4	3.4–5.5	1	
Regular	9.0	7.9–10.2	2.07	1.64–2.61^a^
Poor	27.0	24.1–30.2	6.21	5.03–7.67^a^
Number of chronic diseases^f^				
One or none	5.8	4.7–7.2	1	
Two or more	14.7	13.2–16.5	2.54	2.05–3.15^a^
Difficulty performing BADL^g^				
No	5.8	4.9–6.8	1	
Yes	28.1	25.2–31.1	4.87	4.06–5.83^a^

^a^ p < 0.001

^b^ p < 0.01

^c^ p < 0.05

^d^ Prevalence ratio.

^e^ Estimated 95% confidence intervals.

^f^ History of medical diagnosis of hypertension, diabetes, heart disease, chronic lung disease, stroke, arthritis, asthma, cancer, and kidney disease.

^g^ Difficulty in performing one of the following basic activities of daily living (BADL): bathing, dressing, eating, using the toilet, getting out of bed, crossing a room on the same floor.

Table 3 presents the results of the multivariate analysis. Positive and statistically significant associations were observed for age (PR = 1.69 for the 60–69 age group and PR = 3.49 for those 70 years or older), worse self-rated health (PR = 1.65 for reasonable and PR = 3.17 for poor or very poor), had two or more chronic diseases (PR = 1.34), and had difficulty performing BADL (PR = 2.68). Negative associations were observed for living with a partner (PR = 0.78) and educational level (PR = 0.80, 0.72 and 0.51 for 4–7, 8–11 and 12 years or more, respectively).

**Table 3:** Results of the multivariate analysis of factors associated with frailty. The Brazilian Longitudinal Study of Aging (ELSI-Brazil), 2015-2016.

Variable	Adjusted PR^d^	95%CI^e^
Age (years)		
50–59	1	
60–69	1.69	1.37–2.09^a^
70 or older	3.49	2.82–4.32^a^
Lives with a partner		
No	1	
Yes	0.78	0.67–0.91^b^
Education (years of schooling)		
0–3	1	
4–7	0.80	0.66–0.97^c^
8–11	0.72	0.55–0.93^c^
12 or more	0.51	0.28–0.93^c^
Self-rated health		
Good	1	
Regular	1.65	1.34–2.05^a^
Poor	3.17	2.56–3.93^a^
Number of chronic diseases^f^		
Uma ou none	1	
Two or more	1.34	1.08–1.66^b^
Difficulty performing BADL^g^		
No	1	
Yes	2.68	2.23–3.22^a^

^a^ p < 0.001

^b^ p < 0.01

^c^ p < 0.05

^d^ Prevalence ratio adjusted by gender and income sufficiency.

^e^ Estimated 95% confidence intervals.

^f^ History of medical diagnosis of hypertension, diabetes, heart disease, chronic lung disease, stroke, arthritis, asthma, cancer, and kidney disease.

^g^ Difficulty in performing one of the following basic activities of daily living (BADL): bathing, dressing, eating, using the toilet, getting out of bed, crossing a room on the same floor.

**Figure 2:**
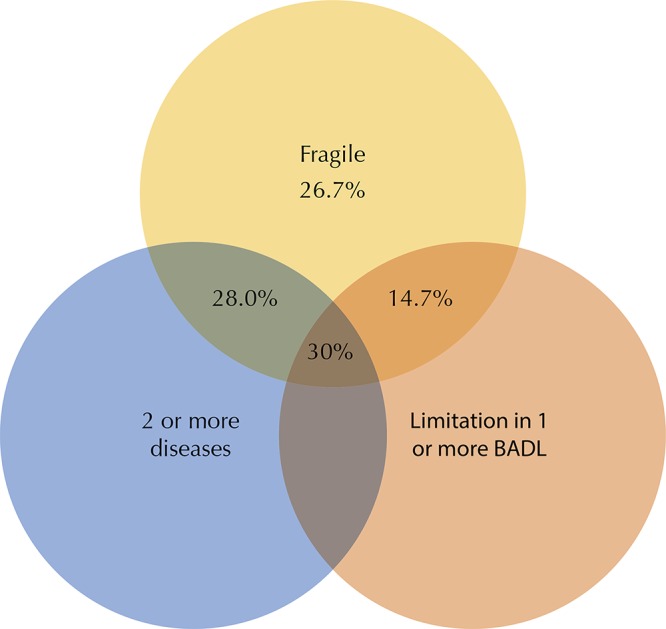
Venn diagram showing the overlap between frailty, chronic diseases and limitations to perform basic activities of daily living (BADL) among older Brazilians. The Brazilian Longitudinal Study of Aging (ELSI-Brazil), 2015–2016.

Figure 2 is the Venn diagram, which shows the overlap between frailty, chronic diseases and difficulty to perform BADL. Of the participants with frailty, 28.0% had two or more chronic diseases; 14.7% had limitations to perform BADL; and 26.7% did not have any of these conditions.

The additional analysis shows that the estimated prevalence of frailty, using imputed data to compensate for the information loss of 9% of those eligible for the study, was similar (9.5%; 95%CI 8.4–10.6) to that without imputation previously shown. It is noteworthy that this same analysis showed that the factors associated with frailty are the same as presented in Table 3 (data not shown).

## DISCUSSION

This was the first study to estimate the prevalence of frailty in the Brazilian population aged 50 years or older. The results show a prevalence of 9% of frailty in this population. This prevalence increases with age and reaches 20.9% among those aged 70 years or older. Frailty was associated with less education and different health conditions. In addition, it was observed that about a quarter of the participants presented frailty in the absence of multimorbidity or limitations to perform BADL.

The prevalence of frailty varies markedly among populations. The comparison between the studies should be done with caution because of the different definitions used and the age brackets considered in each of them. In this study, prevalence rates were estimated for different ages, in order to allow comparisons with other investigations, using similar criteria to define the outcome. The prevalence of frailty in this analysis for the 65 years or older age group (16.2%) was similar to that observed in SHARE (The Survey of Health, Aging and Retirement in Europe) conducted in 10 European countries (17.0%)^6^, but higher than in the FIBRA study, conducted in seven Brazilian cities, which identified prevalence rates between 7.7% and 10.8%^2^. The prevalence found in this analysis was lower than that observed in other Latin American countries (19.6%)^3^, but higher than that observed in the SABE study (8.5%), conducted in the city of São Paulo^12^.

Frailty is one of the major syndromes associated with aging^19^ because as people age, they accumulate deficiencies in various physiological systems and become increasingly vulnerable to disease complications. With age, there’s an increase in the likelihood of neuromuscular changes, neuroendocrine dysregulation, and dysfunctions in the immune system. Thus, the likelihood of the development of disabilities and of chronic diseases onset also increases^1^ ^,^ ^4^ ^,^ ^20^. These conditions, as well as frailty, undergo the cumulative effects of risks present throughout life, associated, for example, with age and gender^1^ ^,^ ^4^. In the present study, there was a positive association between frailty and age, but there was no association with gender.

The results of this study corroborate the relationship between frailty and the presence of disabilities and chronic diseases^1^ ^,^ ^4^ ^,^ ^9^. However, as observed in previous studies^1^ ^,^ ^21^, our results show a significant proportion (26.7%) of individuals with frailty do not present multimorbidity, nor limitations to perform BADL. This proportion is comparable to those found in the United States (26.6%)^1^ and in Hong Kong (23.1%)^21^. These findings reinforce the hypothesis that frailty is a distinct condition associated with physiological dysregulation and that functional diseases and limitations are not necessarily synonymous with frailty^1^ ^,^ ^20^.

In this analysis, the poor self-rated health was associated with a higher prevalence of frailty, confirming the evidence available in the literature^1^ ^,^ ^4^ ^,^ ^5^. Self-rated health has been used as an important marker for the assessment of frailty since the self-perception of good general health is associated with protective effects on the neurological, immunological and endocrine systems^22^. People who negatively assess their own health usually invest less in self-care, do not practice physical activity, go less to the doctor, have no healthy eating habits and exhibit low psychosocial development. Thus, they are more likely to develop frailty^23^.

The results of this study show that the frailty is associated with lower levels of education. As evidenced in other studies^1^ ^,^ ^5^ ^,^ ^6^ ^,^ ^24^, individuals with lower education had a higher prevalence of frailty. Education is a social determinant of health often used in inequity analyses. The low level of education in Brazil compromises access to health and also to better employment and financial conditions, interfering with the style and quality of life of the individual. Consequently, individuals with a lower educational level have higher levels of unhealthy behaviors and develop more chronic diseases, which have an influence on the process of developing frailty^25^.

The association between lower prevalence of frailty and living with a partner calls attention to the importance of social relations and social support for the prevalence^4^ ^,^ ^9^ and the incidence of frailty^26^. Among the possible explanations for the lower prevalence of frailty and the presence of marital relationship, we can highlight the favorable effects of structural support from social relations. Moreover, part of the association between marital status and health comes from positive affection and happiness, and not only from social support^27^. The presence of a partner may also favor health care, such as increasing adherence to medication treatment^28^.

This study has strengths and limitations. The main advantage of the study is its large population base, which allows for the estimation of prevalence rates for the Brazilian population. In addition, frailty was measured using a well-known and standard method, allowing comparisons with other investigations. The limitations are related to the cross-sectional nature of the study, which limits the assessment of causality between associated factors and frailty.

In sum, the prevalence of frailty among Brazilians aged 65 years or older is similar to that observed in the corresponding age group in European countries. Worse health conditions, functional limitation and lower education emerge as the factors most strongly associated with the frailty in this population. The results also show that frailty can occur in the absence of multimorbidity and functional limitations. These findings constitute the first estimate of frailty for the Brazilian population and can provide important information for the planning and implementation of health care interventions and actions to promote better quality of life and active aging among older Brazilians.

## Footnotes

a Fundação Oswaldo Cruz. Brazilian Longitudinal Study of Aging. Rio de Janeiro; c2015 [cited 2017 Nov 28]. Available from: http://elsi.cpqrr.fiocruz.br
